# Is near-infrared spectroscopy a promising predictor for early intracranial hemorrhage diagnosis in the Emergency Department?

**DOI:** 10.1590/1414-431X2023e13155

**Published:** 2024-01-22

**Authors:** O.S. Çınaroğlu, E.S. Bora, H. Acar, C. Arıkan, M. Küçük, S. Kırık

**Affiliations:** 1 Department of Emergency Medicine, Izmir Katip Celebi University, Izmir, Turkey; 2 Department of Emergency Medicine, Izmir Ataturk Training and Research Hospital, Izmir, Turkey; 3 Department of Emergency Medicine, Buca Seyfi Demirsoy Training and Research Hospital, Izmir, Turkey

**Keywords:** Non-invasive optical spectroscopic monitor, Triage, Intracranial hemorrhage, Early prognosis, Headache

## Abstract

Intracranial hemorrhage (ICH) is a serious medical condition that can lead to significant morbidity and mortality if not diagnosed and treated promptly. Early detection and treatment are essential for improving the outcome in patients with ICH. Near-infrared spectroscopy (NIRS) is a non-invasive imaging technique that has been used to detect changes in brain tissue oxygenation and blood flow in various conditions. The aim of this study was to investigate the predictive potential of NIRS for early diagnosis of ICH in patients presenting to the Emergency Department (ED) triage with headache. A total of 378 patients were included in the study. According to the final diagnosis of the patients, 4 groups were formed: migraine, tension-cluster headache, intracranial hemorrhage and intracranial mass, and control group. Cerebral NIRS values “rSO_2_” were measured at the first professional medical contact with the patient. The right and left rSO_2_ (RrSO_2_, LrSO_2_) were significantly lower and the rSO_2_ difference was significantly higher in the intracranial hemorrhage group compared to all other patient groups (P<0.001). The cut-off values determined in the receiver operating characteristics (ROC) analysis were RrSO_2_ ≤67, LrSO_2_ ≤67, and ΔrSO_2_ ≥9. This study found that a difference of more than 9 in cerebral right-left NIRS values can be a non-invasive, easy-to-administer, rapid, and reliable diagnostic test for early detection of intracranial bleeding. NIRS holds promise as an objective method in ED triage for patients with intracranial hemorrhage. However, further research is needed to fully understand the potential benefits and limitations of this method.

## Introduction

The increasing number of patients in emergency departments (EDs) has led to the need for new and effective methods to quickly and accurately identify critically ill patients during first examination (1,2). Emergency physicians and nurses typically use vital signs to differentiate critical patients from others and assign triage categories (Red, Yellow, Green), directing patients with abnormal vital signs to areas where they can receive faster intervention. However, for some complaints, such as headaches, there is a need for accessible and objective parameters that can be used in addition to vital signs. Headache is among the most important complaints, accounting for 0.5-4.5% of all ED visits (3). Most headache cases present to the ED with primary headaches, and approximately half are diagnosed with migraine or other headache types (4). Headaches, which can occur at almost any age, can be due to vascular causes such as thrombosis, hemorrhage, or critical diseases secondary to infections causing neuronal damage without any other neurodegenerative symptoms. However, identifying primary headache types according to the International Headache Society (IHS) criteria during triage is not practical (5-7). No objective parameters are available to categorize headaches during prehospital or initial hospital visits other than consciousness status and vital signs.

Regional cerebral oxygen saturation (rSO_2_) is a noninvasive method for measuring cerebral oxygen saturation at the patient's bedside, with the advantages of being precise, instantaneous, and fast. Near-infrared spectroscopy (NIRS) is based on the principle that near-infrared light can penetrate tissues and be absorbed by oxyhemoglobin and deoxyhemoglobin, which forms the basis of regional cerebral oxygen saturation measurement. Cerebral oximeters in this device provide an average value related to oxygenation in these compartments (8). In the past, NIRS has been reported to detect silent periods of cerebral ischemia and intracranial bleeding and may play an important role in preserving brain function (9). Although the use of NIRS devices has increased in recent years in anesthesia, neurosurgery, and cardiac surgery intensive care units, there is currently no routine and practical use in EDs (10,11).

In this study, we aimed to determine the validity of NIRS in detecting intracranial bleeding during the first examination of patients presenting to the ED with headache or blunt head trauma complaints, using a practical, fast, and noninvasive measurement method during triage evaluation.

## Material and Methods

### Study design

This study was a single-center, prospective observational study. Ethics committee approval for the study was obtained from the non-interventional clinical research ethics committee of Izmir Katip Çelebi University affiliated to the hospital (22/12/2022; number 0580).

### Settings

The study was conducted at Atatürk Training and Research Hospital, which is a tertiary education and research hospital located in an urban area with a population of approximately 4,500,000, with approximately 400,000 patients admitted to the ED each year.

### Participants

#### Inclusion criteria

The population of the study consisted of patients aged 18 years and older who presented to the ED with a headache or blunt head trauma, had stable vital signs (blood pressure, pulse rate, respiratory rate, and oxygen saturation), were conscious, fully co-operative and orientated, had a Glasgow Coma Scale (GCS) of 15, and were planned to undergo a brain computed tomography (CT).

#### Exclusion criteria

Patients under 18 years of age, pregnant women, patients with unstable vital signs (blood pressure, pulse rate, respiratory rate, and oxygen saturation), patients with GCS <15, patients with concomitant amnesia, patients with nausea or vomiting associated with headache, patients with known intracranial space-occupying lesions, patients with a history of cranial surgery, patients with a history of intracranial hemorrhage, patients with a history of ischemic stroke, and patients with high-energy trauma were excluded. High-energy traumas can be classified as either open or closed injuries resulting from the application of force (such as missiles, traffic accidents, crushing or blasting injuries, or falls from heights). These traumas impact the body surface and transmit a significant amount of kinetic energy, leading to extensive tissue damage (12). In addition to the cases in the study, 90 healthy volunteers were included in the control group. No other procedure other than vital signs and NIRS measurement was performed in the control group.

### Data collection and processing

Written informed consent was obtained from those who fulfilled the inclusion criteria and agreed to participate in the study. Demographic characteristics (age and gender), triage code (green, yellow, red), and vital signs (blood pressure, pulse rate, respiratory rate, and oxygen saturation) were recorded on the case data form. Another investigator carried out the NIRS measurement using a daily calibrated NIRS device (Root with O3 Regional Oximetry, Masimo Corporation, USA) following the manufacturer's recommended practices. Two adult adhesive sensors (≥40 kg) placed on the right and left frontal regions of the forehead were used for measurement (13). The difference between the regional tissue oxygen saturation (rSO_2_) values measured at the right and left frontal region and the rSO_2_ values measured from both sensors was recorded on the case report form. Patients were divided according to CT results into groups migraine, tension-cluster headache, intracranial hemorrhage and intracranial mass, and control.

### Outcome measures

rSO_2_ values from the right (RrSO_2_) and left (LrSO_2_) frontal regions were compared and the difference between the rSO_2_ values from both regions was calculated.

### Study size

Since there is no similar study in the literature, the effect size was used when calculating the sample size. Considering the effect size of 0.30, the sample size was determined as 177 with a 95% confidence interval and a 5% margin of error (G*Power 3.1 package program, Heinrich-Heine-Universität Düsseldorf).

However, because we planned to subdivide the patients in the headache group according to the underlying etiology, 378 patients were included in the study: 288 patients in the headache group and 90 patients in the control group.

### Statistical analysis

The data were analyzed using SPSS Statistics version 16 (IBM, USA). Numeric data are reported as numbers, percentages, and means±SD. The Shapiro-Wilk test determined whether the data were normally distributed. A one-way ANOVA test was used for multiple group comparisons. The Tukey test was used to determine which group(s) were significantly different in the multiple group analysis. With the Hosmer and Lemeshow test, since the P value (sig) was greater than 0.05, the model of the study was found to be adequate. Receiver operating characteristics (ROC) analysis was performed to evaluate the sensitivity and specificity of NIRS. The results are reported with a 95% confidence interval. P<0.05 was considered statistically significant.

## Results

A total of 378 cases were included in the study, 153 (40.5%) male and 225 (59.5%) female. According to age groups, 21 individuals (5.6%) were between 18-24 years of age, 222 (58.7%) were between 25-44 years of age, 108 (28.6%) were between 45-64 years of age, and 27 (7.1%) were over 65 years of age. After triage, 18 (4.7%) cases were assigned a red code, 126 (33.3%) cases a yellow code, and 234 (62%) cases a green code (Table 1).

**Table 1:** Patient sociodemographic characteristics and triage codes.

	n (%)
Total number of patients	378 (100)
Gender	
Male	153 (40.5%)
Female	225 (59.5%)
Age group	
18-24	21 (5.6%)
25-44	222 (58.7%)
45-64	108 (28.6%)
65 and above	27 (7.2%)
Triage code	
Red	18 (4.7%)
Yellow	126 (33.3%)
Green	234 (62%)

In the comparative analysis between groups, statistically significant differences between the control, headache (tension-type, cluster-type), migraine, intracranial hemorrhage, and intracranial mass groups were observed for RrSO_2_ and LrSO_2_, as well as for the difference between RrSO_2_ and LrSO_2_ (P<0.001) (Table 2). In the *post hoc* analysis, RrSO_2_ and LrSO_2_ were significantly lower in the intracranial hemorrhage group than in the control, migraine, headache, and intracranial mass groups. Additionally, the ΔrSO_2_ in the intracranial hemorrhage group was significantly higher than in all other patient groups (P<0.001).

**Table 2:** Mean NIRS values and standard deviations according to the etiology of headaches of the 5 groups.

Etiology	Number	Mean	SD	P
RrSO_2_				
Control	90	70	1.9	<0.001
Headache, other	110	69	3.7	
Migraine	95	67	3.1	
Intracranial hemorrhage	65	61	10.6	
Intracranial mass	18	69	2.5	
LrSO_2_				
Control	90	69	2.2	<0.001
Headache, other	110	69	6.1	
Migraine	95	69	2.7	
Intracranial hemorrhage	65	62	8.9	
Intracranial mass	18	69	2.7	
ΔrSO_2_ (|RrSO_2_-LrSO_2_|)				
Control	90	2.25	1.3	<0.001
Headache, other	110	3.71	2.23	
Migraine	95	2.52	1.59	
Intracranial hemorrhage	65	6.24	4.38	
Intracranial mass	18	3.33	1.41	

One-way ANOVA was used for statistical analyses. NIRS: near-infrared spectroscopy; RrSO_2_ and LrSO_2_: right and left regional cerebral oxygen saturation.

**Figure 1:**
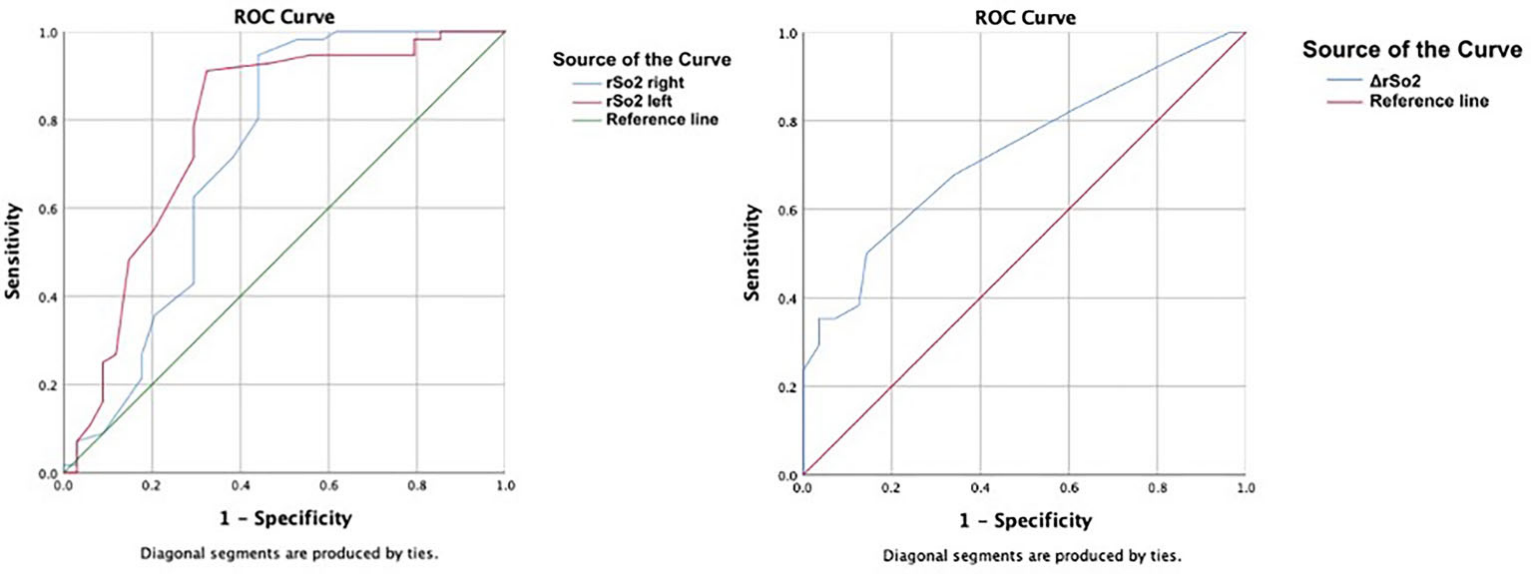
ROC analysis of sensitivity and specificity for near-infrared spectroscopy (NIRS) values measured at the right and left frontal region. rSO_2_: regional cerebral oxygen saturation.

A ROC analysis was performed to evaluate the effectiveness of NIRS analysis in detecting intracranial hemorrhage. The area under the curve (AUC) for all three parameters was strong and similar in detecting hemorrhage (0.721, 0.782, 0.728) (Figure 1). The cut-off values determined in the ROC analysis were RrSO_2_ ≤67, LrSO_2_ ≤67, and ΔrSO_2_ ≥9, and their sensitivity, specificity, positive predictive value (PPV), and negative predictive value (NPV) are presented in Table 3.

**Table 3:** ROC analysis of the frontal NIRS values measured from the right and left and the sensitivity and specificity for the difference in values (ΔrSO_2_) between them.

	AUC	Sensitivity	Specificity	PPV	NPV	P
RrSO_2_ ≤67	0.721	62%	71%	57	75	0.002
LrSO_2_ ≤67	0.782	71%	79%	67	81	<0.001
ΔrSO_2_ ≥9	0.728	23%	100%	100	68	<0.001

NIRS: near-infrared spectroscopy; RrSO_2_ and LrSO_2_: right and left regional cerebral oxygen saturation; AUC: area under the curve; PPV: positive predictive value; NPV: negative predictive value.

## Discussion

This study found that the ΔrSO_2_ (|RrSO_2_- LrSO_2_|) value was predictive for detecting intracranial hemorrhage based on primary headache causes. ΔrSO_2_ can be used as a noninvasive, easy-to-apply, rapid, and, most importantly, reliable diagnostic test for intracranial hemorrhage in the ED with 100% specificity and 100% PPV.

NIRS is a method that has been studied by various clinics for its success in showing cerebral tissue oxygenation and is successful in different intracranial events (10,11). Although CT and magnetic resonance imaging are still the gold standard in cases of headache with suspected brain hemorrhage or infarction according to the literature, NIRS is used as an auxiliary and monitoring test between these devices as it provides less information (14,15). However, there are insufficient studies regarding its diagnostic value in suspicious intracranial events in EDs. This study is one of the rare studies evaluating the use of NIRS in EDs. In this study, contrary to the literature, we aimed to investigate whether NIRS can be used as an effective device in the differential diagnosis of headaches in the ED.

In previous studies, it has been reported that the normal rSO_2_ range in healthy adult volunteers in room air is 71.2±3.9% (16,17). Although most studies indicate that the average rSO_2_ value falls within the normal range of 55 to 75%, individual variability has also been emphasized (18). In our study, the rSO_2_ ratios in the control group of 90 patients were 70 and 69% for the right and left sides, respectively, indicating that the standardization of our study is similar to other studies.

Lazaridis et al. (19) have reported that the first 6 h of intracranial hemorrhage are critical, and rapid diagnosis and treatment are important to avoid irreversible nerve damage. In this regard, procedures performed in EDs come to the fore. Based on the results of our study, we believe that NIRS measurements at the initial contact with patients presenting in the ED with headache complaints are valuable for excluding intracranial hemorrhage.

In a study conducted by Hennes et al. (16) with 212 cases, signal changes were detected in 181 patients in NIRS evaluation performed before CT. Its sensitivity and specificity were found to be 0.96 and 0.29, respectively. Another similar study showed that NIRS values in traumatic brain injury cases in the ED are a good indicator for making operative decisions (20). Our study supports our hypothesis that there is a significant signal change in NIRS parameters measured at triage and/or examination areas in patients presenting with headaches compared to other forms of presentation for intracranial hemorrhage.

According to the ROC analysis, rSO_2_ ≤67 had a predictive value for bleeding (sensitivity 62-71%, specificity 71-79%). However, it can be seen that ΔrSO_2_ ≥9 is a stronger indication for intracranial hemorrhage (sensitivity 23%, specificity 100%). However, ΔrSO_2_ values less than nine do not rule out intracranial hemorrhage. The ΔrSO_2_ value has not been studied thoroughly in previous research. This study is the first to identify a parameter that can detect intracranial hemorrhage at the initial presentation in the ED.

This study demonstrated that NIRS can predict the presence of intracranial bleeding with high accuracy and is more sensitive than traditional methods for early detection. Previous studies have also shown that NIRS can predict intracranial bleeding in head trauma patients (20-22). In our study, we observed stimulatory and sensitive signs in intracranial bleeding patients. At the same time, no significant differences were found in NIRS values among patients with isolated headache, migraine, and intracranial mass groups.

Some studies have reported reduced blood oxygenation levels and altered cerebral blood flow patterns in certain brain regions during migraine attacks compared to periods without headaches (23,24). In contrast, our study did not find any statistical differences in rSO_2_ values among patients with a previous diagnosis of migraine who presented to the ED with a migraine attack, or in the control group and in patients with tension-type headache and intracranial masses.

Intracranial masses are one of the causes of headache. Studies report that huge masses that increase intracranial pressure change NIRS values (25-27). NIRS gives an idea about the size of the mass and the surgical decision in such cases. In this study, the NIRS values of the patients who presented with headaches and were found to have incidental intracranial mass were not at levels alerting the emergency physician; they gave similar results as the migraine, headache, and control groups. This situation is related to the incidental detection of the mass and, therefore, the size of the mass.

### Limitations

The first limitation of this study is that it was a single center study. In addition, the information on diagnosis and bleeding localization (right, left, or bilateral) was not described in detail. In future studies, exact right and left rSO_2_ measurements and measurement of the bleeding area may give more precise results.

Different statistical models may provide more sensitive and specific results and thus strengthen the justification for the use of NIRS.

### Conclusion

This study found that a difference of more than 9 in cerebral right-left NIRS values can be a noninvasive, easy-to-administer, rapid, and reliable diagnostic test for early detection of intracranial bleeding. NIRS is a promising objective method in ED triage of patients with intracranial hemorrhage. However, further research is needed to fully understand its potential benefits and limitations.

## References

[1] Torelli P, Campana V, Cervellin G, Manzoni GC (2010). Management of primary headaches in adult Emergency Departments: a literature review, the Parma E.D. experience and a therapy flow chart proposal. Neurol Sci.

[2] Morgenstern LB, Huber JC, Luna-Gonzales H, Saldin KR, Grotta JC, Shaw SG (2001). Headache in the emergency department. Headache.

[3] Kengne UIM, Tegueu CK, Mankong DS, Mbede M, Tene UG, Moifo B (2020). Clinical predictors of significant intracranial computed tomography scan findings in adults experiencing headache disorder. Pan Afr Med J.

[4] Stevenson RJ, Dutta D, MacWalter RS (1998). The management of acute headache in adults in an acute admissions unit. Scott Med J.

[5] Relja G, Granato A, Capozzoli F, Maggiore C, Catalan M, Pizzolato G (2005). Nontraumatic headache in the emergency department: a survey in the province of Trieste. J Headache Pain.

[B06] Headache Classification Committee of the International Headache Society (2004). The International Classification of Headache Disorders, 2nd edition. Cephalalgia.

[7] Fiesseler FW, Kec R, Mandell M, Eskin B, Anannab M, Riggs RL (2002). Do ED patients with migraine headaches meet internationally accepted criteria?. Am J Emerg Med.

[8] Ferrari M, Quaresima V (2012). A brief review on the history of human functional near-infrared spectroscopy (fNIRS) development and fields of application. Neuroimage.

[9] Hayashi K, Yamada Y, Ishihara T, Tanabe K, Iida H (2022). Comparison of regional cerebral oxygen saturation during one-lung ventilation under desflurane or propofol anesthesia: a randomized trial. Medicine (Baltimore).

[10] Peters J, Van Wageningen B, Hoogerwerf N, Tan E (2017). Near-infrared spectroscopy: a promising prehospital tool for management of traumatic brain injury. Prehosp Disaster Med.

[11] Holmgaard F, Vedel AG, Rasmussen LS, Paulson OB, Nilsson JC, Ravn HB (2019). The association between postoperative cognitive dysfunction and cerebral oximetry during cardiac surgery: a secondary analysis of a randomized trial. Br J Anaesth.

[12] Jovanović M, Janjić Z, Marić D (2002). Principles of management of high-energy injuries of the leg [in Croatian]. Med Pregl.

[13] Lee JH, Song IS, Kang P, Ji SH, Jang YE, Kim EH (2022). Validation of the Masimo O3™ regional oximetry device in pediatric patients undergoing cardiac surgery. J Clin Monit Comput.

[14] Trehan V, Maheshwari V, Kulkarni SV, Kapoor S, Gupta A (2018). Evaluation of near-infrared spectroscopy as a screening tool for detecting intracranial hematomas in patients with traumatic brain injury. Med J Armed Forces India.

[15] Rindler RS, Allen JW, Barrow JW, Pradilla G, Barrow DL (2020). Neuroimaging of intracerebral hemorrhage. Neurosurgery.

[16] Hennes HJ, Lott C, Windirsch M, Hanley DF, Boor S, Brambrink A, Dick W (1997). Noninvasive detection of intracerebral hemorrhage using near-infrared spectroscopy (NIRS). Proceed SPIE.

[17] Ehara N, Hirose T, Shiozaki T, Wakai A, Nishimura T, Mori N (2017). The relationship between cerebral regional oxygen saturation during extracorporeal cardiopulmonary resuscitation and the neurological outcome in a retrospective analysis of 16 cases. J Intensive Care.

[18] Dix LML, van Bel F, Lemmers PMA (2017). Monitoring cerebral oxygenation in neonates: an update. Front Pediatr.

[19] Lazaridis C, Rusin CG, Robertson CS (2019). Secondary brain injury: Predicting and preventing insults. Neuropharmacology.

[20] Dagod G, Roustan JP, Bringuier-Branchereau S, Ridolfo J, Martinez O, Capdevila X (2021). Effect of a temporary lying position on cerebral hemodynamic and cerebral oxygenation parameters in patients with severe brain trauma. Acta Neurochir (Wien).

[B21] Peters J, Van Wageningen B, Hoogerwerf N, Tan E (2017). Near-infrared spectroscopy: a promising prehospital tool for management of traumatic brain injury. Prehosp Disaster Med.

[22] Akyol PY, Bayram B, Acerer A, Girgin MC, Yılmaz DÇ, Men S (2016). Comparison of near-infrared spectroscopy and head CT interpretations of the ED patients with minor head injury. Am J Emerg Med.

[23] Pourshoghi A, Danesh A, Tabby DS, Grothusen J, Pourrezaei K (2015). Cerebral reactivity in migraine patients measured with functional near-infrared spectroscopy. Eur J Med Res.

[24] Liboni W, Molinari F, Allais G, Negri E, Grippi G, Benedetto C (2007). Why do we need NIRS in migraine?. Neurol Sci.

[25] Schytz HW, Amin FM, Selb J, Boas DA (2019). Noninvasive methods for measuring vascular changes in neurovascular headaches. J Cerebral Blood Flow Metab.

[B26] Smirniotopoulos JG, Jäger HR, Hodler J, Kubik-Huch RA, von Schulthess GK (2020). Differential diagnosis of ıntracranial masses. Diseases of the brain, head and neck, spine 2020-2023: diagnostic ımaging.

[27] Müller SJ, Henkes E, Gounis MJ, Felber S, Ganslandt O, Henkes H (2023). Non-ınvasive ıntracranial pressure monitoring. J Clin Med.

